# The Anticancer Agent 3,3'-Diindolylmethane Inhibits Multispecies Biofilm Formation by Acne-Causing Bacteria and Candida albicans

**DOI:** 10.1128/spectrum.02056-21

**Published:** 2022-02-02

**Authors:** Yong-Guy Kim, Jin-Hyung Lee, Sunyoung Park, Jintae Lee

**Affiliations:** a School of Chemical Engineering, Yeungnam University, Gyeongsan, Republic of Korea; INTHERES

**Keywords:** antibiofilm, *Cutibacterium acnes*, 3,3'-diindolylmethane, indole-3-carbinol, multispecies biofilms

## Abstract

The Gram-positive anaerobic bacterium Cutibacterium acnes is a major inhabitant of human skin and has been implicated in acne vulgaris formation and in the formation of multispecies biofilms with other skin-inhabiting organisms like Staphylococcus aureus and Candida albicans. Indoles are widespread in nature (even in human skin) and function as important signaling molecules in diverse prokaryotes and eukaryotes. In the present study, we investigated the antibacterial and antibiofilm activities of 20 indoles against *C. acnes*. Of the indoles tested, indole-3-carbinol at 0.1 mM significantly inhibited biofilm formation by *C. acnes* without affecting planktonic cell growth, and the anticancer drug 3,3′-diindolylmethane (DIM) at 0.1 mM (32 μg/mL) also significantly inhibited planktonic cell growth and biofilm formation by *C. acnes*, whereas the other indoles and indole itself were less effective. Also, DIM at 0.1 mM successfully inhibited multispecies biofilm formation by *C. acnes*, S. aureus, and C. albicans. Transcriptional analyses showed that DIM inhibited the expressions of several biofilm-related genes in *C. acnes*, and at 0.05 mM, DIM inhibited hyphal formation and cell aggregation by C. albicans. These results suggest that DIM and other indoles inhibit biofilm formation by *C. acnes* and have potential use for treating *C. acnes* associated diseases.

**IMPORTANCE** Since indoles are widespread in nature (even in human skin), we hypothesized that indole and its derivatives might control biofilm formation of acne-causing bacteria (Cutibacterium acnes and Staphylococcus aureus) and fungal Candida albicans. The present study reports for the first time the antibiofilm and antimicrobial activities of several indoles on *C. acnes*. Of the indoles tested, two anticancer agents, indole-3-carbinol and 3,3′-diindolylmethane found in cruciferous vegetables, significantly inhibited biofilm formation by *C. acnes*. Furthermore, the most active 3,3′-diindolylmethane successfully inhibited multispecies biofilm formation by *C. acnes*, S. aureus, and C. albicans. Transcriptional analyses showed that 3,3′-diindolylmethane inhibited the expressions of several biofilm-related genes including lipase, hyaluronate lyase, and virulence-related genes in *C. acnes*, and 3,3′-diindolylmethane inhibited hyphal formation and cell aggregation by C. albicans. Our findings show that 3,3′-diindolylmethane offers a potential means of controlling acne vulgaris and multispecies biofilm-associated infections due to its antibiofilm and antibiotic properties.

## INTRODUCTION

Cutibacterium acnes, formerly known as Propionibacterium acnes, is a Gram-positive obligate anaerobic bacterium frequently found in sebaceous follicles of human skin. Although generally considered a human skin commensal, C. acnes secretes proteases, lipases, hemolysins, and porphyrins that degrade host tissues and is also considered as opportunistic causative pathogen of acne vulgaris (1).

*C. acnes* readily forms biofilm on plastic, glass, steel, titanium, silicone, and other surfaces, and its biofilms are often resistant to conventional antibiotics and host immune systems (2). Also, *C. acnes* is often found in multispecies biofilms with skin-colonizing organisms such as Staphylococcus aureus and Candida albicans (3–5). *C. acnes* biofilms are composed of DNA, proteins, and glycosyl residues (6). Although the effects of medium, culture time, and surface type on the biofilm characteristics of several *C. acnes* isolates have been studied *in vivo* and *in vitro* (7), the mechanism responsible for biofilm formation remains unclear despite the attempts made to control the formation of opportunistic *C. acnes* biofilms (8–10).

Indole is synthesized from tryptophan by tryptophanase by many gut and environmental bacteria, and is present in animal intestinal tracts and human skin (11). Indole and its derivatives act as interspecies and interkingdom signaling molecules and play important roles in bacterial pathogenesis and eukaryotic immunity (12, 13). In particular, indole controls quorum sensing and biofilm formation of its own species and in other bacterial species (12). However, several indole derivatives have more potent antimicrobial and antibiofilm activities than indole such as 3-indolylacetonitrile, 7-hydroxyindole, 7-benzyloxyindole, methylindoles, iodoindoles, fluoroindoles, chloroindoles against Escherichia coli (14, 15), Pseudomonas aeruginosa (15), Paenibacillus alvei (16), S. aureus (17), C. albicans (18), Acinetobacter baumannii (19), Serratia marcescens (20), Vibrio parahaemolyticus (21), and others.

Skin-colonizing *C. acnes* is almost certainly accustomed to indole containing environments, as indole constitutes nearly 30% of the volatile headspace of sweat (22). Hence, we investigated the antibacterial and antibiofilm potentials of 20 natural or synthetic indole derivatives against *C. acnes*. To investigate how indoles influence biofilm development, we used confocal laser scanning microscopy (CLSM), scanning electron microscopy (SEM), and quantitative real-time PCR (qRT-PCR), and extracellular polymeric substance (EPS) production, hyphal, and combinatorial antibiotic assays. In addition, we investigated the antibiofilm activities of 3,3′-diindolylmethane (the more active indole) against multispecies biofilms of *C. acnes*, S. aureus, and C. albicans. The present study reports for the first time the antibiofilm and antimicrobial activities of several indoles on multispecies microbes including acne-causing bacteria and C. albicans.

## RESULTS

### Antimicrobial and antibiofilm activities of indoles against *C. acnes*.

**FIG 1:**
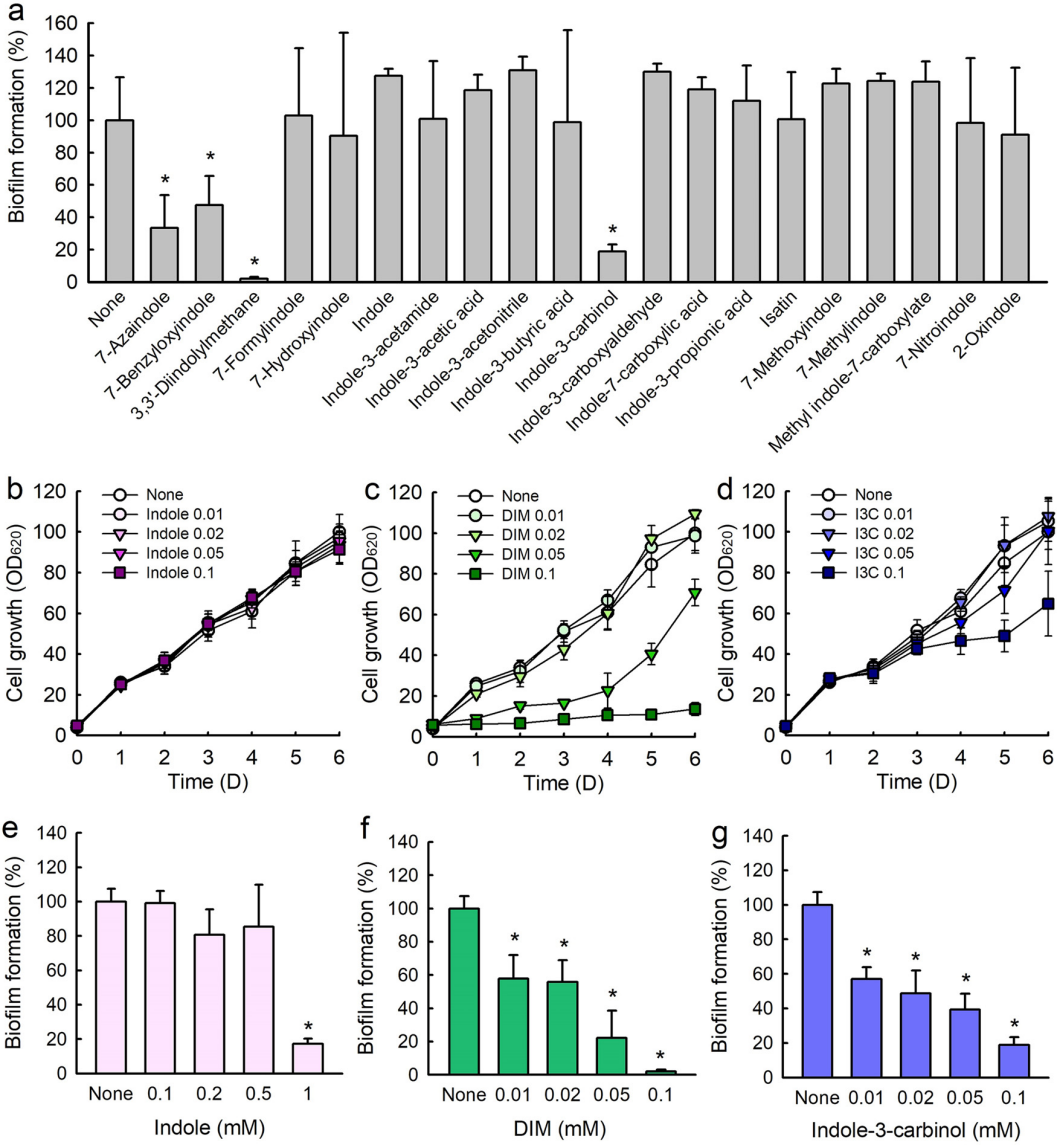
Antibiofilm activities of indole derivatives against *C. acnes*. Screening of *C. acnes* biofilm formation in the presence of indole derivatives at 0.1 mM (a). Cell growths in the presence of indole (b), DIM (c), and indole-3-carbinol (d). Antibiofilm activities of indole (e), DIM (f), and indole-3-carbinol (g). Error bars indicate standard deviations. *, *P < *0.05 versus nontreated controls.

To assess antibiofilm properties against *C. acnes*, 20 indole derivatives at a concentration of 0.1 mM were initially screened in 96-well plates (Table 1). Among the indoles tested, 7-azaindole, 7-benzyloxyindole, 3,3′-diindolylmethane (DIM), and indole-3-carbinol at 0.1 mM significantly inhibited biofilm formation by *C. acnes*, while indole and other indoles were less effective (Fig. 1a).

**TABLE 1:** The indole derivatives examined and their MICs against *C. acnes*

Indoles	MIC (mM)	Indoles	MIC (mM)
7-Azaindole	>5	Indole-3-carbinol	>5
7-Benzyloxyindole	>5	Indole-3-carboxyaldehyde	>5
3,3′-Diindolylmethane (DIM)	0.1	Indole-7-carboxylic acid	>5
7-Formylindole	>5	Indole-3-propionic acid	>5
7-Hydroxyindole	>5	Isatin	5
Indole	5	7-Methoxyindole	>5
Indole-3-acetamide	>5	7-Methylindole	1
Indole-3-acetic acid	>5	Methyl indole-7-carboxylate	5
Indole-3-acetonitrile	5	7-Nitroindole	2
Indole-3-butyric acid	5	2-Oxindole	>5

An MIC assay was used to examine the antibacterial profiles of the 20 indoles against *C. acnes*. While MICs of most indoles were > 5 mM, the MICs of DIM, 7-methylindole, and 7-nitroindole were 0.1 mM, 1.0 mM, and 2.0 mM, respectively, after 6 days of culture (Table 1). Also, planktonic growth curves showed that indole up to 0.1 mM did not inhibit cell growth (Fig. 1b), DIM at 0.1 mM completely inhibited cell growth (Fig. 1c), and indole-3-carbinol at 0.1 mM partially inhibited cell growth (Fig. 1d). These results show DIM and indole-3-carbinol inhibited *C. acnes* biofilm formation partially due to their antibacterial activity.

### Indole-3-carbinol, DIM, and antibiotics inhibited *C. acnes* biofilm formation.

Detailed biofilm study showed that indole, indole-3-carbinol, and DIM concentration dependently inhibited *C. acnes* biofilm formation (Fig. 1e to g). For example, indole at 1 mM (117 μg/mL) significantly inhibited *C. acnes* biofilm formation (Fig. 1e). This is the first report that indole has antibiofilm activity against *C. acnes*. Most notably, DIM at 0.1 mM (32 μg/mL) almost abolished biofilm formation (Fig. 1f), while indole-3-carbinol dose-dependently inhibited *C. acnes* biofilm formation but less potently than DIM (Fig. 1g).

**FIG 2:**
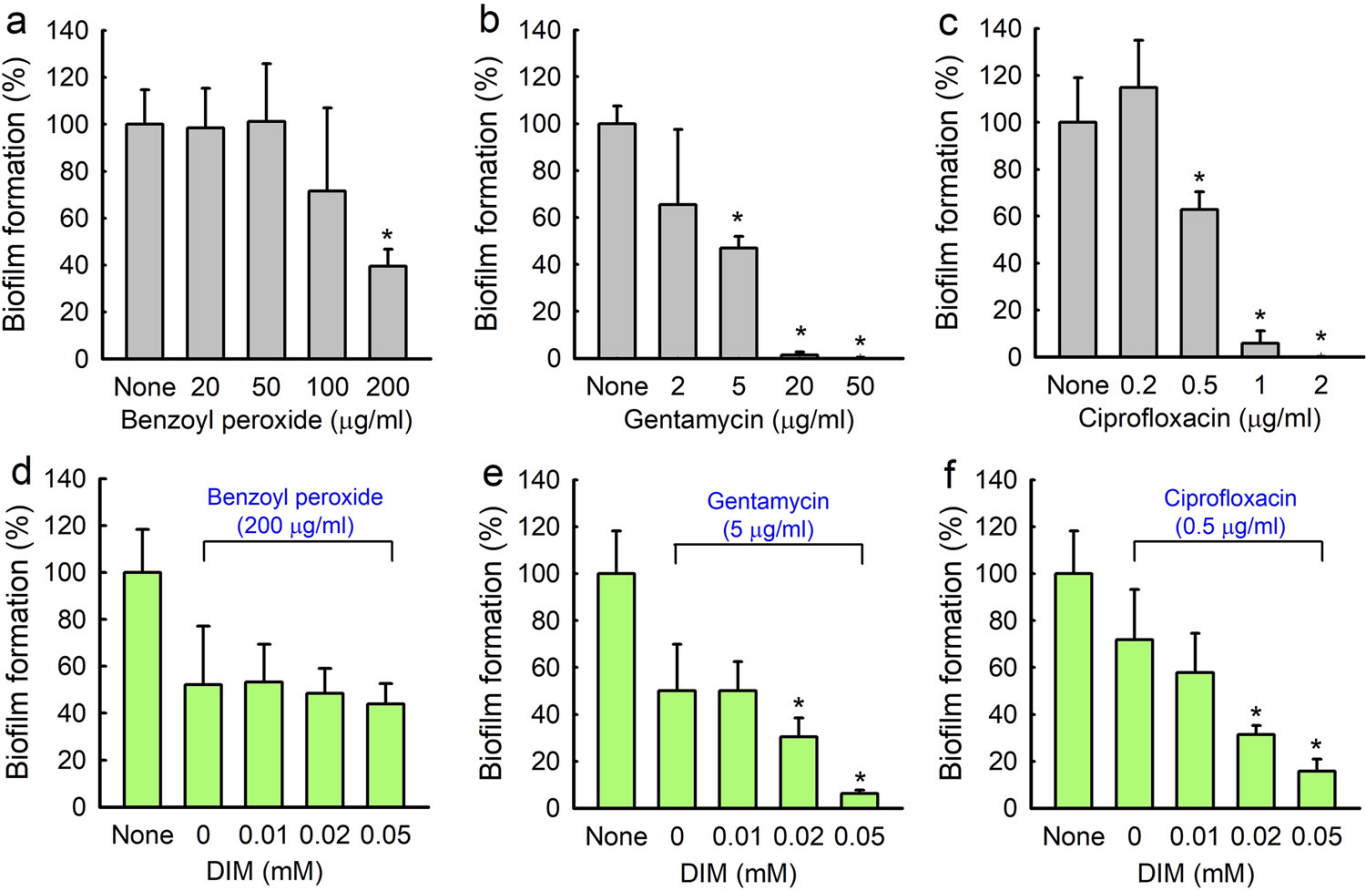
*C. acnes* biofilm inhibition by antibiotics in the presence or absence of DIM. Biofilm inhibitions by benzoyl peroxide (a), gentamicin (b), and ciprofloxacin (c) were measured after incubation for 6 days in 96-well plates at 37°C under anaerobic conditions. Antibiofilm activities of benzoyl peroxide (d), gentamicin (e), and ciprofloxacin (f) in the presence of 3,3′-diindolylmethane (DIM) were measured after incubation under identical conditions. Error bars indicate standard deviations. *, *P < *0.05 versus nontreated controls.

The antibiofilm activity of DIM was compared with those of three antibiotics, namely, benzoyl peroxide, gentamicin, and ciprofloxacin, which also dose-dependently inhibited *C. acnes* biofilm formation (Fig. 2). While benzoyl peroxide, which is commonly used to treat acne, was less effective at inhibiting *C. acnes* biofilm formation than DIM, gentamicin and broad-spectrum antibiotic ciprofloxacin were more effective at inhibiting *C. acnes* cell growth and biofilm formation. For example, benzoyl peroxide at 200 μg/mL inhibited biofilm formation by 60% (Fig. 2a) with MIC 400 μg/mL, which concurs with a recent report (23). Gentamycin at 20 μg/mL inhibited biofilm formation by 98% (Fig. 2b) with MIC 100 μg/mL, and ciprofloxacin at 1 μg/mL inhibited biofilm formation by 94% (Fig. 2c) with MIC 5 μg/mL.

### Combinatory antibiofilm efficacies of DIM and antibiotics against *C. acnes*.

Antibiofilm activities were enhanced when DIM and antibiotics were used in combination. Concentrations of antibiotics resulting in 40 ∼ 60% biofilm reduction were selected for this study. Gentamycin at 5 μg/mL reduced biofilm formation by 53%, whereas treatment with gentamicin (5 μg/mL) and DIM (0.05 mM) reduced biofilm formation by 93% (Fig. 2e). Also, ciprofloxacin at 0.5 μg/mL reduced biofilm formation by 37%, whereas treatment with ciprofloxacin (0.5 μg/mL) and DIM (0.05 mM) reduced biofilm formation by 84% (Fig. 2f). However, DIM did not enhance the efficacy of benzoyl peroxide (Fig. 2d). Fractional inhibitory concentration index (FICI) was calculated to check synergistic effect of DIM and antibiotics. FICI values are 1.0 for gentamicin and 1.0 for ciprofloxacin, respectively. Hence, it appeared that the combinatory effects were additive and not synergistic.

### Antibiofilm activities of DIM against C. albicans and S. aureus biofilms.

**FIG 3:**
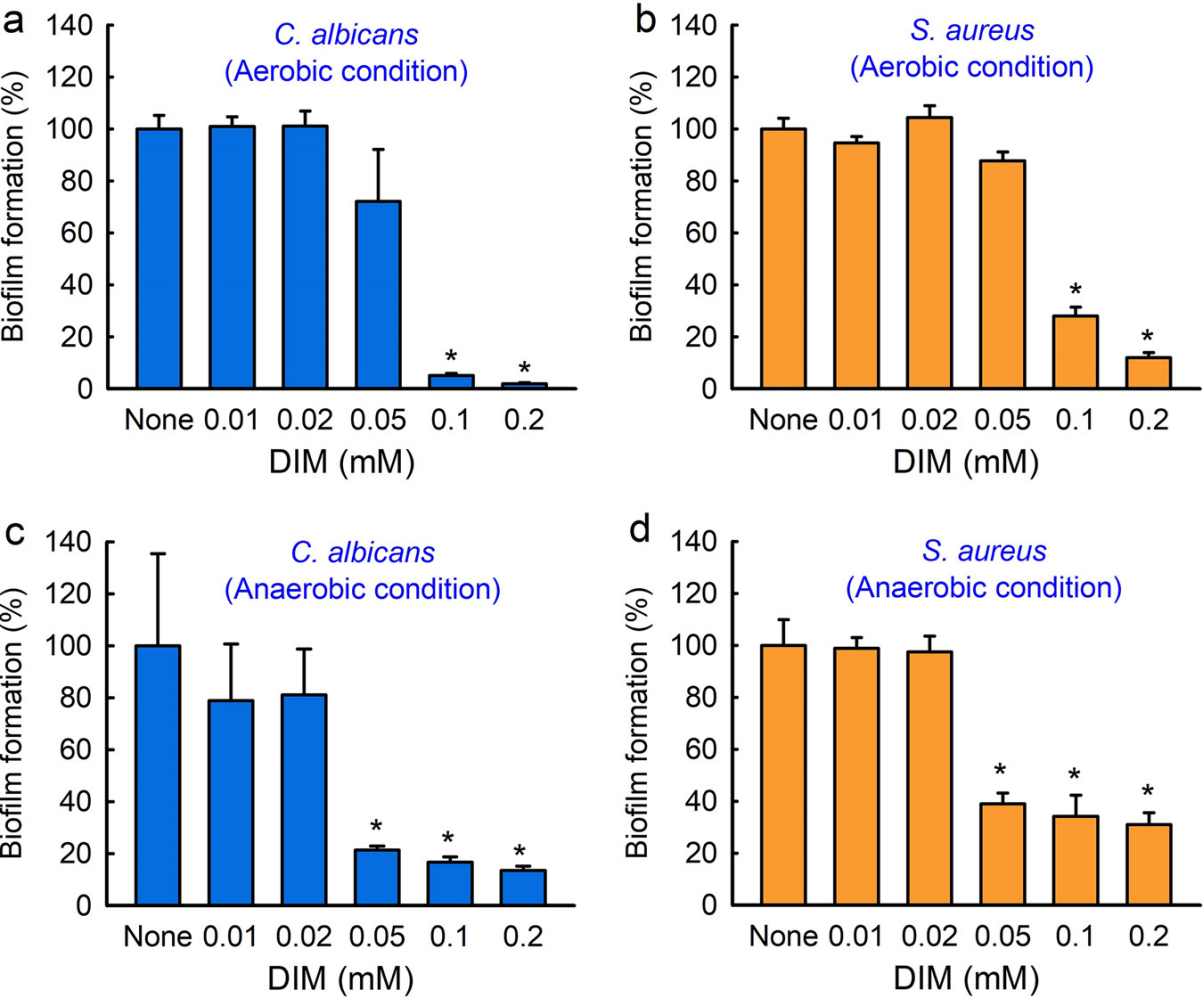
Antibiofilm activities of 3,3′-diindolylmethane (DIM) against C. albicans and S. aureus under anaerobic and aerobic conditions. Biofilm formation by C. albicans (a) and S. aureus (b) was measured after incubation for 24 h in 96-well plates at 37°C under aerobic conditions. Biofilm formations by C. albicans (c) and S. aureus (d) were measured after incubation for 6 days in 96-well plates at 37°C under anaerobic conditions. Error bars indicate standard deviations. *, *P < *0.05 versus nontreated controls.

*C. acnes* often forms biofilms on skin with other microorganisms like S. aureus or C. albicans (3, 4), and these biofilms tend to be more tolerant of antimicrobials (24). Hence, we investigated the inhibitory effects of DIM on S. aureus or C. albicans biofilms under aerobic and anaerobic conditions. Biofilm formations by C. albicans and S. aureus were dose-dependently inhibited by DIM after culture for 1 day under aerobic conditions (Fig. 3a and b). For example, DIM at 0.1 mM inhibited C. albicans biofilm formation by 94% (Fig. 3a) and inhibited S. aureus biofilm formation by 71% under aerobic conditions (Fig. 3b). Furthermore, DIM significantly inhibited biofilm formations by C. albicans and S. aureus after culture for 6 days under anaerobic conditions (Fig. 3c and d). More specifically, DIM at 0.1 mM inhibited C. albicans biofilm formation by 83% (Fig. 3c) and inhibited S. aureus biofilm formation by 65% (Fig. 3d). The MICs of DIM against C. albicans and S. aureus were both > 1 mM, respectively, which suggest that the antimicrobial activity of DIM was partially responsible for its antibiofilm activity.

### Microscopic observations of *C. acnes* biofilm inhibition by DIM.

**FIG 4:**
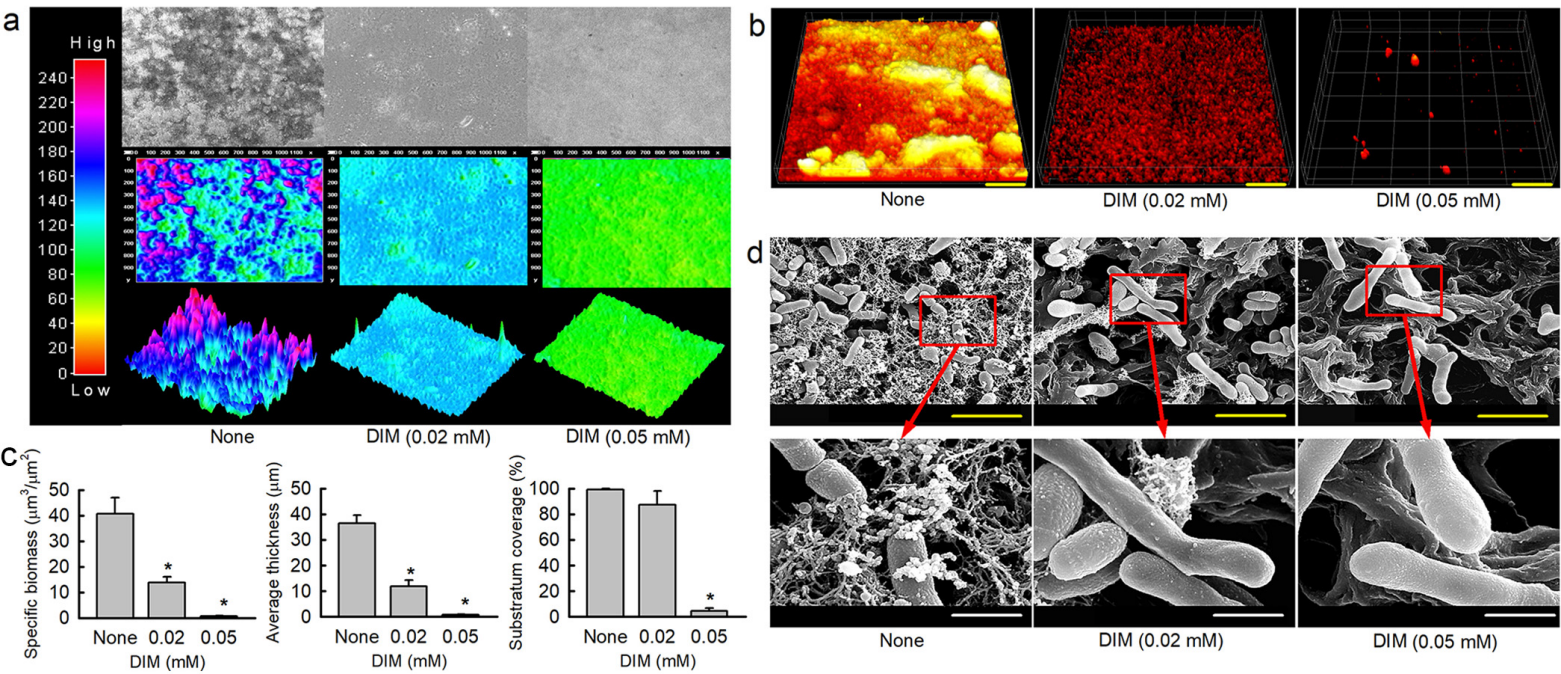
Antibiofilm effects of DIM on *C. acnes*. The antibiofilm activity of DIM against *C. acnes* was determined after culture for 6 days under anaerobic conditions. Re-created *C. acnes* biofilm color-coded 2-D and 3-D images after culture in the presence of DIM (0, 0.02, or 0.05 mM) (a), and CLSM images (b) and COMSTAT analysis results (c) of *C. acnes* biofilm inhibition by DIM. Scale bars in panel b represent 100 μm. *, *P < *0.05 versus nontreated controls. SEM images of *C. acnes* biofilms formed in the presence or absence of DIM (0, 0.02, or 0.05 mM) (d). Yellow and white scale bars represent 3 μm and 750 nm, respectively in panel d. None: nontreated control.

Biofilm inhibition was analyzed using a live cell imaging system and by CLSM and SEM. 3-D color mesh plots revealed that DIM at 0.02 or 0.05 mM inhibited biofilm formation by *C. acnes* (Fig. 4a). CLSM and COMSTAT analysis better revealed biofilm changes (Fig. 4b and c). Nontreated *C. acnes* formed dense biofilms (thickness > 40 μm and almost 100% surface coverage), and the presence of DIM at 0.02 or 0.05 mM significantly reduced biofilm densities and thicknesses (Fig. 4c). Specifically, biofilm biomass, thickness, and substrate coverage were reduced by > 88, 87, and 95% versus untreated controls, respectively, in the presence of 0.05 mM DIM (Fig. 4c).

SEM analysis showed that DIM decreased the production of EPS and reduced *C. acnes* cell densities in biofilms. Intriguingly, DIM treatment increased cell length, indicating inhibition of cell division (Fig. 4d). More specifically, *C. acnes* cell sizes increased by 240% (3.0 ± 0.3 μm) and 252% (3.2 ± 0.2 μm) in the presence of 0.02 or 0.05 mM DIM, respectively, as compared with non-treated controls (1.25 ± 0.4 μm) (Fig. 4d). Interestingly, indole and indole-3-carbinol at 0.05 mM also decreased EPS production and increased *C. acnes* cell sizes (Fig. S1 in the supplemental material).

### Antibiofilm activities of DIM against multispecies biofilms of *C. acnes*, C. albicans, and S. aureus.

**FIG 5:**
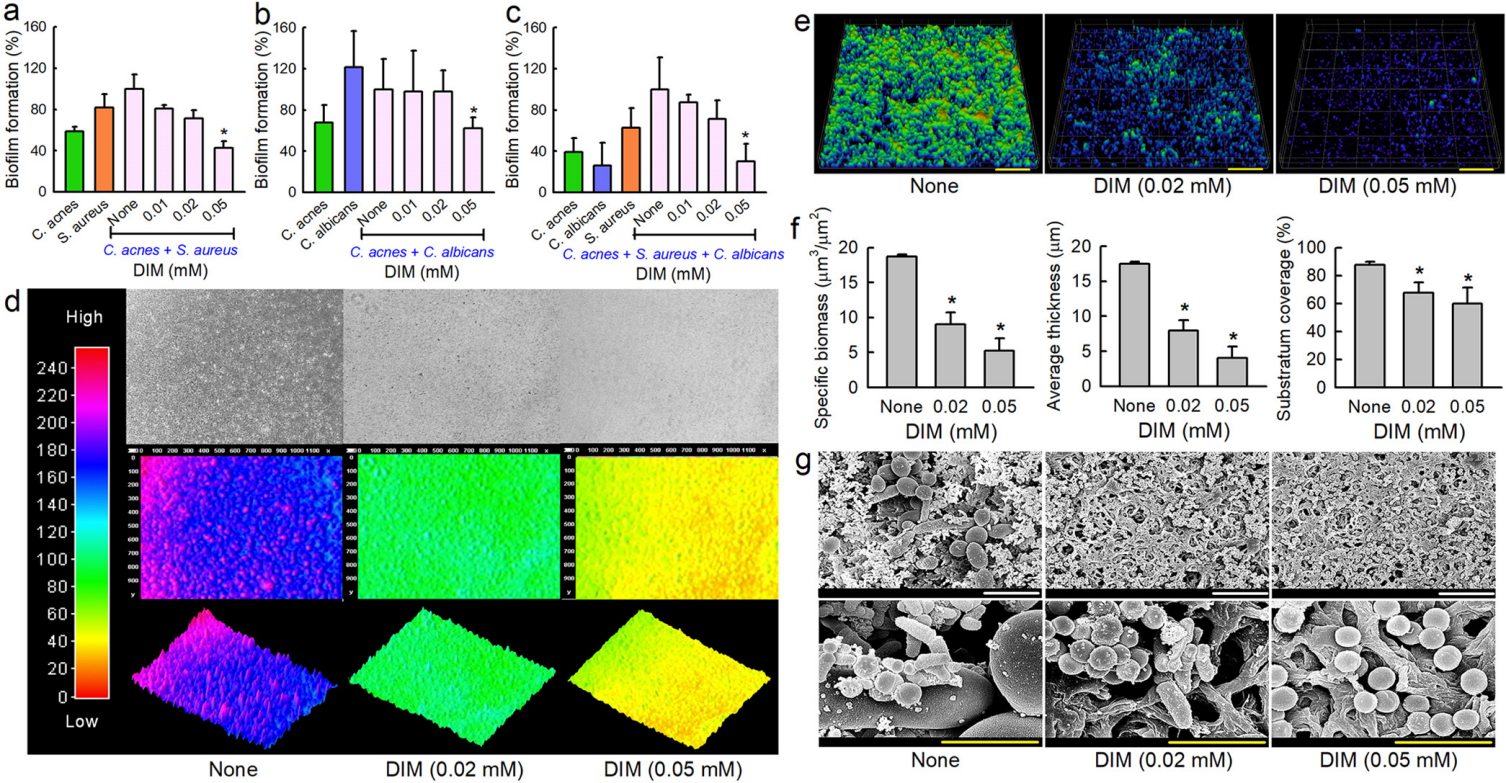
Effects of DIM on polymicrobial biofilms. Antibiofilm activities of DIM against polymicrobial biofilms of *C. acnes* and S. aureus (a), *C. acnes* and C. albicans (b), and *C. acnes*, C. albicans, and S. aureus (c) biofilms after culture under anaerobic conditions. Color-coded 2-D and 3-D images of 3-component *C. acnes*, C. albicans, and S. aureus biofilms cultured in the presence of DIM (d), CLSM images (e), and COMSTAT analysis results (f) of three species biofilms cultured in the presence of DIM. Scale bars represent 100 μm. *, *P < *0.05 versus nontreated controls. SEM images of three species biofilms formed in the presence or absence of DIM (g). White and yellow scale bars represent 10 and 3 μm, respectively in panel g. None: nontreated control.

Since DIM effectively inhibited biofilm formation by *C. acnes* (Fig. 1), C. albicans (Fig. 3c), and S. aureus (Fig. 3d), we investigated the antibiofilm activities of DIM against polymicrobial biofilms containing two or three species. We optimized media and inoculum to form biofilms from two different combinations of two species and one combination of three species: *C. acnes* and S. aureus (Fig. 5a) in 1:1 combination of Reinforced Clostridium Media (RCM) and Luria-Bertani (LB); *C. acnes* and C. albicans (Fig. 5b) in a 1:1 mixture of RCM and Potato Dextrose Broth (PDB); and *C. acnes*, S. aureus, and C. albicans (Fig. 5c) in a 1:1:1 mixture of RCM, LB, and PDB. As was expected, DIM dose-dependently inhibited biofilm formation in all cases (Fig. 5a to c). Notably, DIM at 0.05 mM inhibited three-species biofilm formation by 69% after culture for 6 days under anaerobic conditions (Fig. 5c). To the best of our knowledge, this is the first report of three species biofilms of *C. acnes*, C. albicans, and S. aureus.

Multispecies biofilms were observed using a live cell imaging system and by CLSM and SEM. All three techniques showed DIM inhibited three species biofilms (Fig. 5d to g). They formed biofilms of thickness ∼ 19 μm with almost 100% surface coverage, but DIM at 0.02 or 0.05 mM dose-dependently reduced biofilm densities and thicknesses (Fig. 5f). On the other hand, three species biofilms were substantially less dense and thinner than a single *C. acnes* biofilm (Fig. 4b). SEM visualized the presence of *C. acnes*, C. albicans, and S. aureus in mixed biofilms (Fig. 5g). In the nontreated control, C. albicans formed pseudohyphae and yeast cells that were much larger than the round cells of S. aureus cells or the rod-type cells of *C. acnes*. DIM treatment at 0.02 mM prevented the adhesion of most C. albicans cells, and few *C. acnes* cells were observed among S. aureus cells. Notably, DIM at 0.05 mM significantly reduced the production of EPS in three species biofilms, and only few numbers of S. aureus cells remained while C. albicans cells and *C. acnes* cells were not shown.

### DIM reduced EPS production in *C. acnes* biofilms.

**FIG 6:**
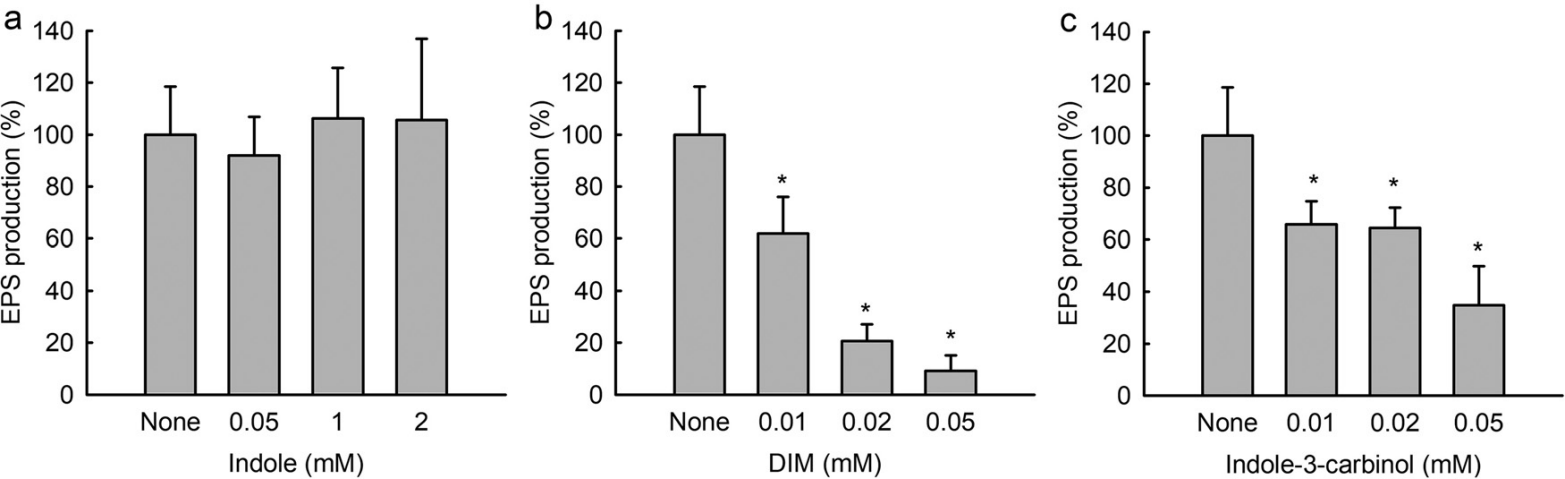
Effects of indole, DIM, and indole-3-carbinol on EPS production by *C. acnes*. Extracellular polymeric substance production by *C. acnes* in the presence of indole (a), DIM (b), or indole-3-carbinol (c). *, *P < *0.05 versus nontreated controls.

EPS production is a hallmark of biofilm formation as it protects cells from environmental challenges. EPS production by *C. acnes* was measured in the presence of indole, DIM, and indole-3-carbinol. While indole at concentrations up to 2 mM did not affect EPS production (Fig. 6a), DIM and indole-3-carbinol dose-dependently reduced EPS production in *C. acnes* (Fig. 6b and c), and DIM at 0.05 mM reduced EPS production in *C. acnes* by 90%. These results suggest that the antibiofilm activity of DIM is partially due to the inhibition of EPS production.

### DIM and indole-3-carbinol inhibited hyphal growth and cell aggregation by C. albicans.

**FIG 7:**
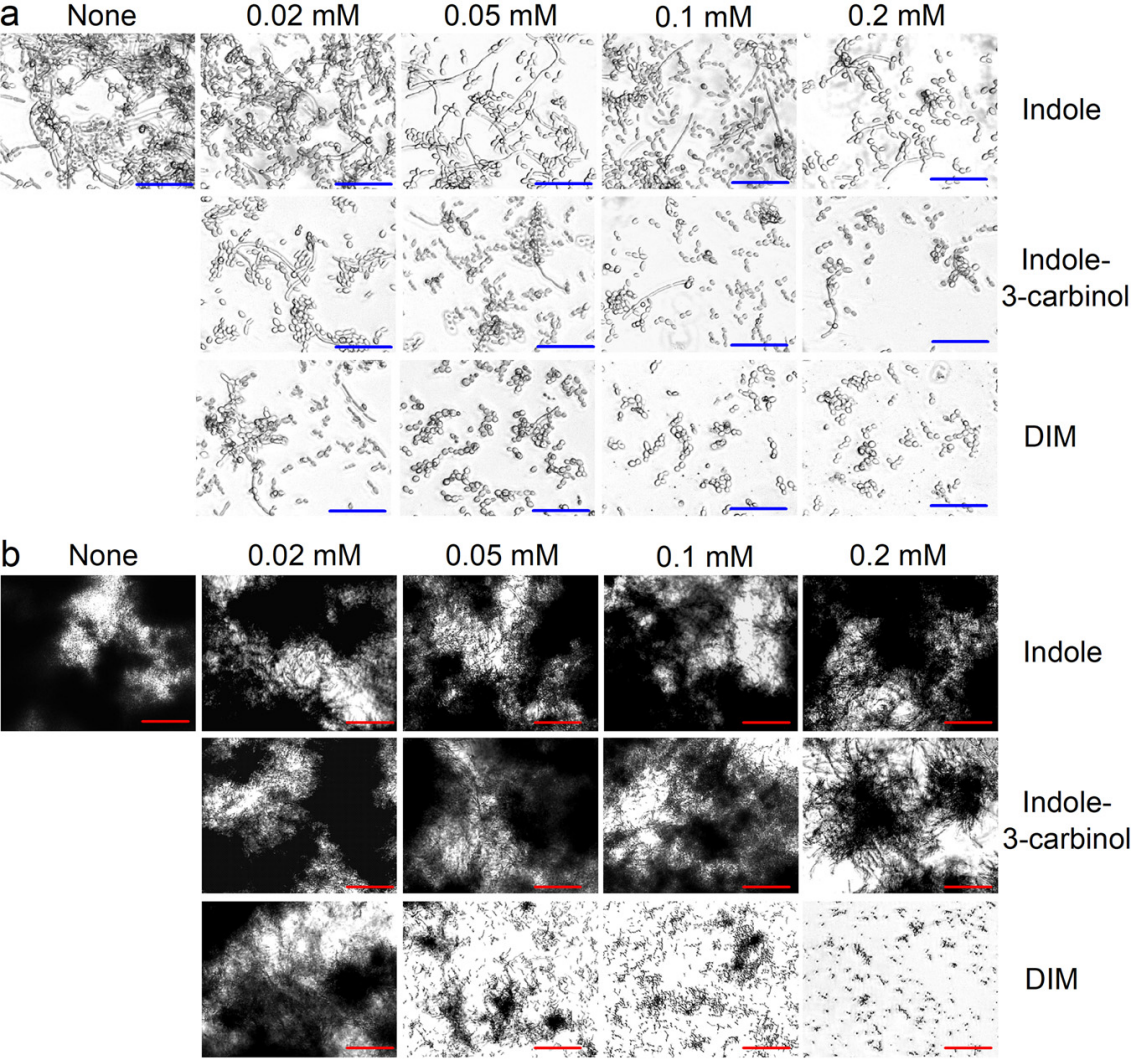
DIM inhibited hyphal filamentation and aggregation by C. albicans. Inhibition of filamentous hyphal growth in PDB medium (a) and cell aggregation in RPMI medium containing 10% fetal bovine serum (b). Hyphae were visualized after incubation for 24 h at 37°C without agitation. The scale bars represent 100 μm in panels a and b. None: nontreated control.

Dimorphic switching of yeast cells to hyphal cells and cell aggregation are prerequisites of C. albicans biofilm maturation (25). To study the effect of indoles on C. albicans morphology, we used a microscope and a cell aggregation assay. In untreated C. albicans, hyphae and large cell aggregations entangled by hyphae were observed after 24 h. Treatments with DIM or indole-3-carbinol at 0.1 mM all substantially suppressed hyphal formation; yeast and pseudohyphae cells were mostly observed (Fig. 7a). Indole at concentrations up to 0.2 mM did not affect this dimorphic switching, but DIM at 0.05 mM clearly inhibited hyphal formation and cell aggregation (Fig. 7b). These results indicate DIM potently inhibits C. albicans biofilm formation by inhibiting hyphal formation and cell aggregation.

### DIM-induced changes in gene expressions in *C. acnes*.

**FIG 8:**
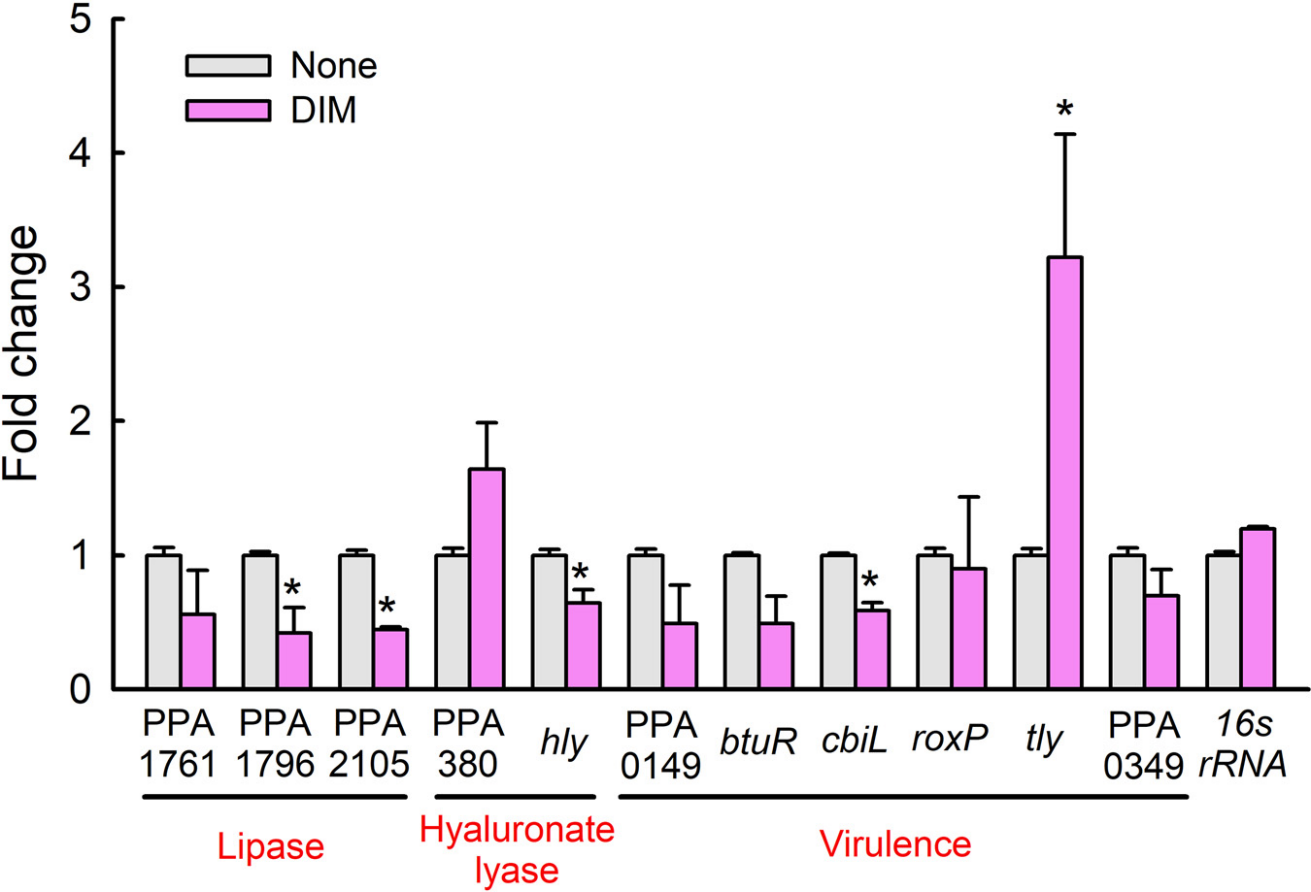
Relative transcriptional profiles of *C. acnes* cells treated with DIM. *C. acnes* cells grown for 3 days were treated with DIM at 0.1 mM for 24 h without shaking. Transcriptional profiles were analyzed by qRT-PCR. Fold changes represent transcriptional changes of treated versus untreated *C. acnes*. The experiment was performed in duplicate (six qRT-PCRs were performed per gene). *, *P < *0.05 versus nontreated controls (None).

qRT-PCR was used to investigate the effect of DIM on the expressions of 11 biofilm- and virulence-related genes in *C. acnes*. Overall, DIM modulated the expression of several lipase genes, hyaluronate lyase genes, and virulence-related genes (Fig. 8), while the expression of the housekeeping gene (*16s rRNA*) was unchanged. Notably, the expressions of lipase genes (PPA1796 and PPA2105), the hyaluronate lyase gene (*hly*), and the precorrin-2 C(20)-methyltransferase (*cbiL*) gene were downregulated while the expression of hemolysin (*tly*) was upregulated 3-fold by DIM at 0.1 mM.

## DISCUSSION

Acne vulgaris is a common chronic skin disease that results in psychological and social problems in adolescents, and it is generally accepted that biofilm formation by skin microorganisms like *C. acnes*, S. aureus, and C. albicans aggravate acne vulgaris (2, 26). In this study, we aimed to identify a biofilm inhibitor against biofilms formed by *C. acnes*, S. aureus, and C. albicans singly or in combination. DIM was found to be an effective antibiofilm agent, and its action mechanism was partially revealed.

Several indole derivatives have been shown to exhibit antibiofilm activity against various microbes, for example, 3-indolylacetonitrile against enterohemorrhagic E. coli O157:H7 (14), methylindoles (18) or 7‐benzyloxyindole (27) against C. albicans, iodoindoles against A. baumannii (19), several halogenated indoles derivatives against S. marcescens (20), and chloroindoles against V. parahaemolyticus (21). Unlike other indoles in previous studies, in this study, DIM and indole-3-carbinol both efficiently inhibited biofilm formation by *C. acnes*. Notably, microbes respond differently to indoles, and it would appear that a methanol group at the C-3 position of the indole moiety might have been responsible for the antibiofilm activities of DIM and indole-3-carbinol.

**FIG 9:**
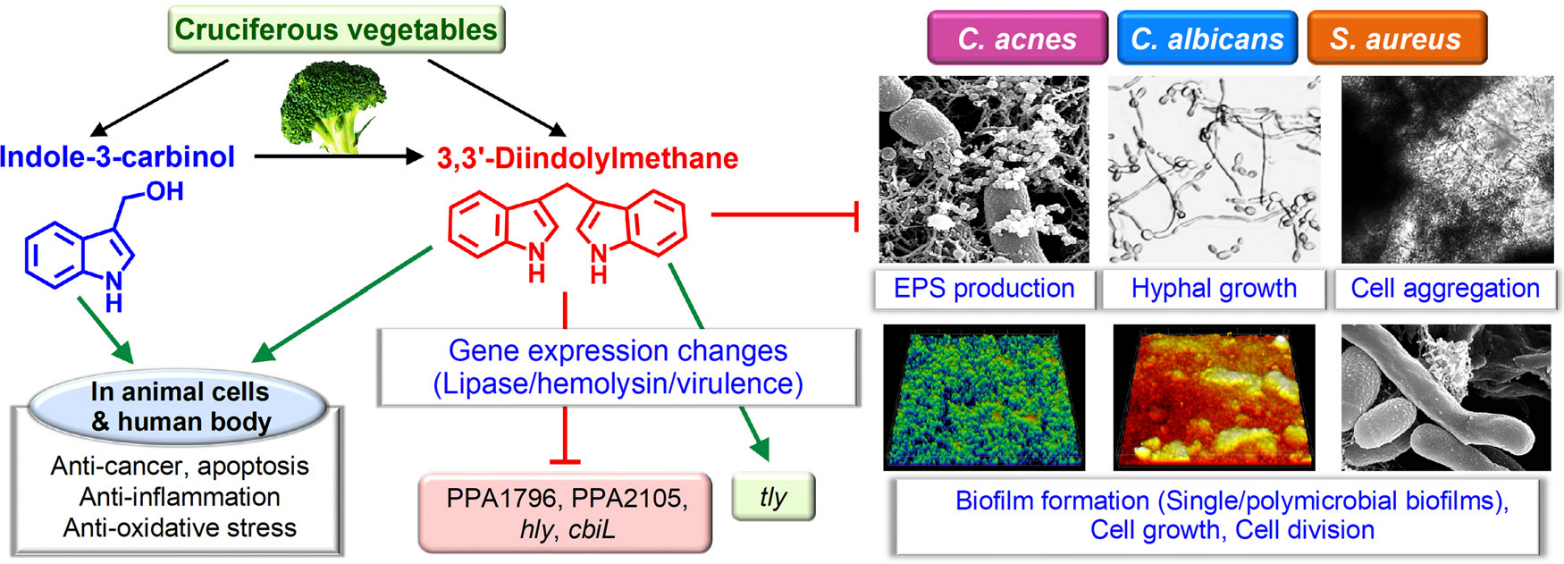
Diagram of the putative functions of indole-3-carbinol and 3,3′-diindolylmethane in single and polymicrobial biofilms.

Most plants have acquired defense mechanisms against environmental microbes, and thus, diverse secondary metabolites represent major pharmaceutical sources for discovery (28, 29). For example, cruciferous vegetables (*Brassica*), such as broccoli, cauliflower, and cabbage, contain various indole derivatives such as indole-3-acetic acid, indole-3-acetonitrile, indole-3-carbinol, and 3,3′-diindolylmethane (DIM) (Fig. 9). Indole-3-acetic acid and indole-3-acetonitrile are growth hormones (auxins) (30), and indole-3-acetonitrile has been reported to inhibit E. coli O157:H7 biofilm formation and to act as an antivirulence compound against P. aeruginosa (14). Also, it was reported that indole-3-carbinol and DIM exhibit antimicrobial, antiviral, and anticancer activities (31, 32). DIM is produced from the digestion of indole-3-carbinol in *Brassicas*, and it is currently used to treat respiratory tumors (33). It has been reported that oral dose of DIM at 2 mg/kg/day was tolerated with no significant toxicity (34). Also, the cytotoxicity of DIM was relatively low as EC_50_ of DIM was a range of 230 ∼ 440 μM against three glioblastoma cell lines (35). While its cytotoxicity is inevitable as an anticancer agent, the toxicity of DIM on normal skin cells or skin diseases would be further studied. In this study, DIM shows antimicrobial and antibiofilm activities against *C. acnes*, S. aureus, and C. albicans singly and in combination. Hence, it appears that *Brassicas* utilize several indole derivatives for defense purposes against diverse microbes. Further studies are required to determine the effects of these indoles on other microbes.

Natural occurring biofilms usually consist of multiple microbe species, which means the majority of studies on polymicrobial biofilms are limited, because focus has been placed on the susceptibilities of single microbe biofilms, which are more susceptible than composite biofilms (36). However, this study presents the antibiofilm activities of DIM in mono-, dual-, and three-component biofilm models against Gram-positive *C. acnes*, Gram-positive S. aureus, and fungal C. albicans. As was expected, the antibiofilm mechanisms of DIM appeared to depend on microbe type.

*C. acnes* expresses multiple genes encoding lipases that degrade sebum lipids and release free fatty acids (1), and it has been reported that surface characteristics, biofilm-related genes, adhesive proteins, and lipase activity are involved in *C. acnes* biofilm development and that *C. acnes* contains multiple lipases that degrade sebum lipids (1). Our qRT-PCR study showed that DIM downregulated the expressions of lipase genes (PPA1796 and PPA2105), the hyaluronate lyase (*hly*), and precorrin-2 C(20)-methyltransferase (*cbiL*) genes (Fig. 8). Previously, it was reported that extracellular lipases are associated with biofilm formation by *C. acnes* as biofilm cells produce more extracellular lipases than planktonic cells (37). Hence, it appears that suppressions of these lipase genes by DIM possibly underlie its inhibition of biofilm formation. Additionally, the inhibition of EPS production by DIM (Fig. 6a) supports its biofilm inhibitory effects, since EPS formation is a hallmark of biofilm formation. However, DIM did not affect the hydrophobic characteristics of *C. acnes* cells (Fig. S2 in the supplemental material). More detailed study is required to understand more clearly the mechanism of antibiofilm action.

In C. albicans, the morphogenetic switching of yeast cells to hyphal cells is considered to play an important role in biofilm formation and the pathogenesis of fungal infections (38), and it has been reported that 7-benzyloxyindole (27) and several methylindoles (18) inhibit C. albicans biofilm formation by inhibiting hyphal formation. In the present study, DIM inhibited hyphae and biofilm formation (Fig. 3c and 7a), possibly by reducing the virulence of C. albicans. On the other hand, DIM did inhibit biofilm formation by S. aureus under aerobic and anaerobic conditions (Fig. 3b and d). As was expected, the inhibitory effects of DIM on dual- and three-species biofilms appeared to be more complex (Fig. 5).

Indole-3-carbinol and DIM both induced the apoptosis of human breast cancer cells (39) and human prostate cancer cells (40) and exhibited antioxidative and anti-inflammatory properties (41). Currently, DIM is used to treat recurrent respiratory papillomatosis with tumors in the upper respiratory tracts caused by human papillomavirus (33), and in a preclinical study was well tolerated at a dose of 2 mg/kg/day (34). In the present study, DIM inhibited biofilm formation by *C. acnes* and exhibited potent antimicrobial (Fig. 1f and Table 1) and antibiofilm formation activities by all three microbes (Fig. 5c). Furthermore, DIM and antibiotic treatment in combination enhanced the antibiofilm activities of antibiotics (Fig. 2). These results show DIM, a natural product found in cruciferous vegetables, has potential utility for the treatment of multispecies infections and as a potent antibiofilm agent and antibiotic adjuvant.

## CONCLUSIONS

In conclusion, we report the antimicrobial and antibiofilm activities of a number of indoles, including DIM, against single or composite biofilms produced by *C. acnes*, S. aureus, and/or C. albicans. The antibiofilm activities of DIM appeared to have been due to suppressions of the expressions of lipase genes, antimicrobial activity against *C. acnes*, and inhibition of hyphae development by C. albicans. The study shows DIM offers a potential means of controlling acne vulgaris and multispecies biofilm-associated infections due to its antibiofilm and antibiotic properties.

## MATERIALS AND METHODS

### Strains and chemicals.

*C. acnes* KCCM 41747 (ATCC 6919) (isolated from human facial acne), methicillin-sensitive S. aureus ATCC 6538, and fluconazole-resistant C. albicans strain DAY185 were used in this study. The *C. acnes* strain was cultured on Reinforced Clostridium Media (RCM)-agar plates for colony preparation and in liquid RCM at 37°C under anaerobic conditions (BD GasPak EZ Gas Generating Anaerobic Pouch Systems; Fisher Scientific, Pittsburgh, PA, USA) for all other experiments. The S. aureus strain was cultured in LB medium at 37°C, and the C. albicans strain was cultured in Potato Dextrose Broth (PDB) medium at 37°C.

Twenty indoles, namely, 7-azaindole, 7-benzyloxyindole, 3,3′-diindolylmethane (DIM), 7-formylindole, 7-hydroxyindole, indole, indole-3-acetamide, indole-3-acetic acid, indole-3-acetonitrile, indole-3-butyric acid, indole-3-carbinol, indole-3-carboxyaldehyde, indole-7-carboxylic acid, indole-3-propionic acid, isatin, 7-methoxyindole, 7-methylindole, methyl indole-7-carboxylate, 7-nitroindole, and 2-oxindole were purchased from Sigma-Aldrich (St. Louis, MO, USA), Wako Chemicals Inc. (Richmond, VA, USA) or Combi-Blocks, Inc. (San Diego, CA, USA). Dimethyl sulfoxide (DMSO) was used to dissolve all indoles, and 0.1% (vol/vol) DMSO was used as the negative control; at this concentration, DMSO did not affect bacterial growth or biofilm formation.

### Planktonic cell growth measurements and MIC determinations.

To investigate the effect of indoles on the planktonic cell growth of *C. acnes*, 6 days cultures were reinoculated (dilution 1:50) in the RCM medium and treated with different concentrations of indole (0, 0.1, 0.2, 0.5, 1.0, or 2.0 mM), DIM (0, 0.01, 0.02, or 0.05 mM), or indole-3-carbinol (0, 0.01, 0.02, 0.05, or 0.1 mM) in 96-well plates. Plates were incubated for 6 days at 37°C under anaerobic conditions using anaerobic pouches (Fisher Scientific, Pittsburgh, PA, USA), and cell turbidities were then measured at 620 nm using a Multiskan EX microplate reader (Thermo Fisher Scientific, Waltham, MA, USA).

### Biofilm formation inhibition assay.

Biofilm formation was quantified using crystal violet (Sigma-Aldrich), as previously described (42). Briefly, a 6-day culture of *C. acnes* with an initial OD of 2.3 (CFU ∼10^8^ cells/mL) at 600 nm was diluted 1:50 with RCM medium, distributed in a 96-well plate (300 μL) with or without indoles, and incubated at 37°C under anaerobic conditions for 6 days. After incubation, planktonic cells were discarded, and wells were washed three times with water (300 μL). Attached biofilms in each well were stained with crystal violet (0.1%) for 20 min and washed three times with water, and the crystal violet was extracted with 95% ethanol. Absorbances were measured at 570 nm using a Multiskan EX microplate reader (Thermo Fisher Scientific, Waltham, MA, USA). Results are expressed as the averages of at least 12 replicate wells. Biofilm percentages represent biofilm formation in the presence of each indole compared, expressed as percentages of biofilm formation by untreated controls. Also, the antimicrobial and antibiofilm efficacies of indoles were compared with those of benzoyl peroxide (20, 50, 100, or 200 μg/mL), a well-known antibiotic used to treat acne, gentamicin (2, 5, 20, and 50 μg/mL), and ciprofloxacin (0.2, 0.5, 1.0, and 2.0 μg/mL).

### Assay of biofilm formation by multispecies biofilms of *C. acnes*, S. aureus, and/or C. albicans.

To produce multispecies biofilms of *C. acnes*, S. aureus, and C. albicans, *C. acnes* (59 ± 4 × 10^7^ CFU), S. aureus cells (59 ± 4 × 10^7^ CFU), and C. albicans cells (23 ± 3 × 10^5^ CFU) were inoculated at the same time into a mixed RCM/LB/PDB medium (1:1:1) in 96-well polystyrene plates (total 300 μL/well; SPL Life Sciences, Korea) and incubated at 37°C under anaerobic conditions using anaerobic pouches for 6 days, and then biofilm formation was assayed as described above. For dual species biofilm formation, *C. acnes* and S. aureus or *C. acnes* and C. albicans were inoculated into an RCM/LB (1:1) or a RCM/PDB medium (1:1) in 96-well plates and incubated for 6 days at 37°C under anaerobic conditions.

### Biofilm observations by live imaging microscopy, CLSM, and SEM.

To quantify *C. acnes* biofilms or mixed *C. acnes*/S. aureus/C. albicans biofilms grown in the presence or absence of DIM (0, 0.02, and 0.05 mM), cells (300 μL) were incubated at 37°C under anaerobic conditions using anaerobic pouches for 6 days. After incubation, planktonic cells were removed by washing with PBS three times and biofilms were examined by live imaging microscopy using the iRiS Digital Cell Imaging System (Logos BioSystems, Anyang, Korea) at different magnifications. Biofilm images were reconstructed as color-coded 2-D and 3-D pictures using ImageJ (https://imagej.nih.gov/ij/index.html).

*C. acnes* biofilms or mixed *C. acnes*, S. aureus, and/or C. albicans biofilms were produced on 96-well polystyrene plates (SPL Life Sciences, Pocheon, Korea) in the presence or absence of DIM (0, 0.02, or 0.05 mM) without shaking under anaerobic conditions for 6 days. After incubation, planktonic cells were discarded by washing with water three times, and biofilm cells were treated with carboxyfluorescein diacetate succinimidyl ester, which stains viable cells in biofilms (Invitrogen, Eugene, OR, USA). Biofilm cells on the plate were visualized by a 488 nm Ar laser (emission wavelength 500 to 550 nm) using a CLSM (Nikon Eclipse Ti, Tokyo, Japan). COMSTAT software was used to determine biofilm volumes (μm^3^/μm^2^), mean biofilm thicknesses (μM), and substratum coverages (%) (43). Two independent cultures were used for each experimental sample, and at least 10 random spots were assayed.

SEM was also used to observe multispecies biofilms on nylon membranes, as previously described (44). Briefly, a nylon membrane (Merck Millipore, Burlington, USA) was cut into 0.4 × 0.4 cm pieces and placed in 96-well plates containing single or mixed species grown with or without DIM and incubated for 6 days at 37°C. Cells attached to the nylon membrane pieces were fixed with 2.5% glutaraldehyde and 2% formaldehyde for 20 h, postfixed using OsO_4_, and dehydrated by an ethanol series (50, 70, 80, 90, 95, and 100%) for 30 min each and isoamyl acetate for 20 h. After critical-point drying, cells were examined and imaged using an S-4200 or S-4800 scanning electron microscope (Hitachi, Tokyo, Japan) at a voltage of 15 kV.

### EPS assay.

For EPS quantification, 50 mL cultures of *C. acnes* treated or not treated with indoles at 37°C for 6 days were used. After incubation, cultures were centrifuged at 8,228 × *g* for 10 min and supernatants were discarded. Cell pellets were washed with sterile PBS to remove residual culture supernatants, and then 50 mL of isotonic buffer (10 mM Tris/HCl, pH 8.0, 10 mM EDTA, 2.5% NaCl) was added to each cell pellet, mixed, incubated at 4°C overnight, vortexed for 5 min, and centrifuged at 8,228 × *g* for 10 min. EPS was precipitated from supernatants by adding ice-cold ethanol at a ratio of 1:3 and placing supernatants in a freezer at −20°C overnight. Precipitated EPS was collected by centrifugation at 18,514 × *g* for 30 min at 4°C. The EPS pellets obtained were washed with 70% ethanol, dried, and weighed. Percentage inhibition of EPS production by indoles was calculated by expressing treated EPS weights as percentages of control weights (45).

### Hyphae formation and cell aggregation assay for C. albicans.

To observe filamentous growth and hyphal formation, a microscopic live cell imaging system was used as previously described (46). Briefly, C. albicans cells were reinoculated at 1:50 dilution in 1 mL of PDB medium containing different concentrations (0, 0.02, 0.05, 0.1, or 0.2 mM) of indole, indole-3-carbinol, or DIM and incubated for 24 h at 37°C without shaking. Cells were then visualized using an iRiS Digital Cell Imaging System (Logos BioSystems, Anyang, Korea) according to the manufacturer’s instructions; at least four independent cultures were used. For cell aggregation assessment, C. albicans cells were inoculated at a dilution of 1:50 with overnight culture into 1 mL of hyphal promoting medium (RPMI 1640 without bicarbonate; GE Healthcare Bio-Sciences, Pittsburgh, PA) supplemented with 10% fetal bovine serum (GE Healthcare Bio-Sciences) in 1.7-mL tubes and incubated at 37°C without shaking for 24 h. After incubation, cells were visualized in a bright field under the iRiS Digital Cell Imaging System at ×4 and ×10 magnifications. At least five independent experiments were performed.

### Quantitative real-time PCR (qRT-PCR).

Fifteen mL of *C. acnes* at an initial turbidity of 0.05 at OD_600_ (23 ± 3 × 10^5^ CFU) was inoculated into RCM broth in 15 mL conical tubes and incubated for 3 days at 37°C without agitation under anaerobic conditions. After incubation, cells were incubated with or without DIM (0.1 mM) for an additional 24 h at 37°C without agitation. In order to prevent RNA degradation, RNAlater (RNase inhibitor, Ambion, TX, USA) was used before harvesting cells. Total RNA was extracted and cleaned using a Qiagen RNeasy minikit (Valencia, CA, USA).

qRT-PCR was used to determine the expressions of biofilm- and virulence-related genes, namely, PPA1761, PPA1796, PPA2105, PPA380, *hly*, PPA0149, *btuR*, *cbiL*, *roxP*, *tly*, and PPA0349. The specific primers and the housekeeping gene (*16s rRNA*) used for qRT-PCR are listed in Table S1. The expression of *16s rRNA* was not affected by DIM. The qRT-PCR method used has been previously described (44) and was performed using SYBR Green Master Mix (Applied Biosystems, Foster City, CA, USA) and an ABI StepOne Real-Time PCR System (Applied Biosystems). Analysis of gene expression by qRT-PCR was done by the Applied Biosystems StepOne Real-Time PCR System analysis software. Each gene expression level was normalized by housekeeping gene expressions. At least two independent cultures and four PCRs were used.

### Statistical analysis.

Replication numbers for each experiment are provided in each method, and results are presented as means ± standard deviations. One-way ANOVA followed by Dunnett’s test using SPSS Ver. 23 (Chicago, IL, USA) was performed for the statistical analysis. *P* values of < 0.05 were considered significant. Asterisks in the figures indicate significant changes between treated (none) and untreated samples.
