# Supplementary material for: The Anticancer Agent 3,3'-Diindolylmethane Inhibits Multispecies Biofilm Formation by Acne-Causing Bacteria and Candida albicans
Source: Microbiol Spectr. 2022 Feb 2;10(1):e02056-21. doi: 10.1128/spectrum.02056-21 (PMC8809333; doi:10.1128/spectrum.02056-21)
Supplement: SUPPLEMENTAL FILE 1 — Supplemental material. Download SPECTRUM02056-21_Supp_1_seq14.pdf, PDF file, 0.7 MB [file spectrum02056-21_supp_1_seq14.pdf]

Supplementary materials

Table S1. qRT-PCR primer sequences used for *C. acnes*.

Gene	Description	Primer
**PPA1761**	Lipase	Forward 5'-CCT CAA GGT GTC TGG TCA TC-3'<br>Reverse 5'-GAC TTC GTC GTT TTT CCA GA-3'
**PPA1796**	Lipase	Forward 5'-CTC AAG GTT CGT GAA CGA GT-3'<br>Reverse 5'-GCC ATA GAG CTC CTT GTT GA-3'
**PPA2105**	Lipase	Forward 5'-GAT TTC CTT AGC ACG TGG AG-3'<br>Reverse 5'-GAT GAC GGT GTA GGC GAT AC-3'
**PPA380**	Hyaluronate lyase	Forward 5’-CGC TCT GAA GGA TTC GTC-3’<br>Reverse 5’-GTC GTG CAG GAT ACA CAT GA-3’
hly	Hyaluronate lyase	Forward 5’-CAA CAT CGC CGT GTT TAT TG-3’<br>Reverse 5’-CCC ATG ACG ACG TAG AGG AT-3’
**PPA0149**	Glycosyltransferase	Forward 5'-AGT ACA TGG CTT CCC GAG TG-3'<br>Reverse 5'-CTT GGG ACT CGA AGT TGA GC-3'
btuR	Cobinamide/cobalamin adenosyltransferase	Forward 5'-GGA AGA TGC TCT TCG GGC GCT-3'<br>Reverse 5'-GCC TCA GGG TTC TCC GCA GC-3'
cbiL	Precorrin-2 C(20)-methyltransferase	Forward 5'-GCG CGA GGC AGA CGT GAT CC-3'<br>Reverse 5'-GAC ACC GGA CCT CTC CCG CA-3'
roxP	Radical oxygenase	Forward 5'-GCA TCT AGC CCT CTC ACC AT-3'<br>Reverse 5'-CTG AGA GTC CGG TAG GTG GT-3'
tly	Lysis of red blood cells	Forward 5'-CAG GAC GTG ATG GCA ATG CGA-3'<br>Reverse 5'-TCG TTC ACA AGA CCA CAG TAG C-3'
**PPA0349**	Polysaccharide capsule biosynthesis	Forward 5’-CTT CTT CGT CGA CCA GTT CC-3'<br>Reverse 5’-TCA GCT GTC TCG TCA ACA CC-3'
16s rRNA	16S ribosomal RNA (House keeping gene)	Forward 5'-GGG GCT TAA CCC TGA GCG TGC -3'<br>Reverse 5'-TTC GCT CCC CAC GCT TTC GC-3'

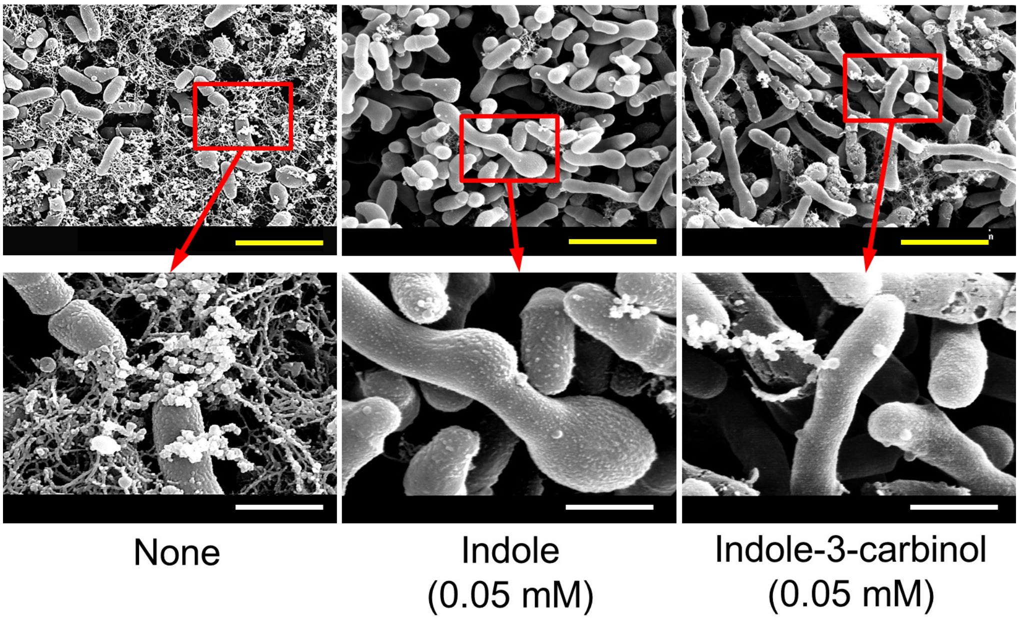

Fig. S1. SEM images of *C. acnes* biofilms formed in the presence or absence of indole and indole-3-carbinol. Yellow and white scale bars represent 3 μm and 750 nm, respectively.

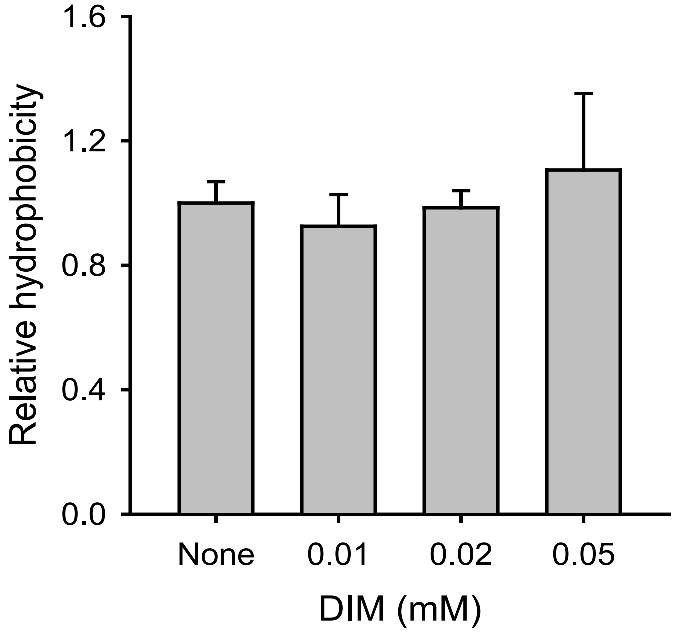

Fig. S2. Effect of DIM on the hydrophobicity of *C. acnes*. The cell surface hydrophobicity of *C. acnes* with or without individual DIM (0.01, 0.02 and 0.05 mM). *C. acnes* was cultured with or without DIM for 6 days at 37°C under the anaerobic condition. After incubation, cell cultures were prepared in sterile PBS at an OD_{600} of ~ 0.5 by a proper dilution and same volume of toluene was added to 3 ml aliquots of cell suspensions and vortexed for 90 s. After vigorous mixing, the toluene phase was completely separated by incubation at room temperature for 10 h. The OD_{600} values of aqueous phases were measured and cell surface hydrophobicity was quantified.
